# Complications Associated with Immunosuppressive Agents in Solid Organ Transplant Recipients: A Nationwide Analysis

**DOI:** 10.3390/jcm14103602

**Published:** 2025-05-21

**Authors:** Ah Young Lee, Jonghyun Jeong, Kyu-Nam Heo, Soyoung Park, Young-Mi Ah, Ji Min Han, Ju-Yeun Lee, Sang Il Min

**Affiliations:** 1 College of Pharmacy and Research Institute of Pharmaceutical Sciences, Seoul National University, Seoul 08826, Republic of Korea; leeah25@snu.ac.kr (A.Y.L.); ksbs1957@snu.ac.kr (J.J.); bogopa8@snu.ac.kr (K.-N.H.); sesthdud2003@snu.ac.kr (S.P.); 2 College of Pharmacy, Yeungnam University, Gyeongsangbuk-do, Gyeongbuk 38541, Republic of Korea; ymah@ynu.ac.kr; 3 College of Pharmacy, Chungbuk National University, Cheongju 28644, Chungcheongbuk-do, Republic of Korea; jmhan@chungbuk.ac.kr; 4 Department of Surgery, Seoul National University Hospital, Seoul 03080, Republic of Korea

**Keywords:** solid organ transplantation, immunosuppression, complications, immunosuppressive agents, post transplantation

## Abstract

**Background:** Immunosuppressive therapies are vital for solid organ transplant (SOT) recipients to ensure graft survival, but long-term use can lead to complications. This study aimed to comprehensively evaluate the complications associated with immunosuppressive agents across different types of major SOTs. **Methods:** In a retrospective cohort study using a national claims database, we analyzed adult SOT recipients who began immunosuppressive therapy from 2007 to 2018. We identified complications such as infections, acute kidney injury, hypertensive emergencies, chronic kidney disease, hypertension, diabetes, dyslipidemia, and osteoporosis. These outcomes were determined through diagnostic codes, medication usage data, and hospital or emergency department visits. **Results:** Among 30,997 transplants with three-year follow up, complication rates varied by transplant type. Pancreatic transplant recipients had the lowest complication rate (225.9 per 1000 patient-years), while lung transplant recipients experienced the highest rate (823.9 per 1000 patient-years). Serious infections and chronic kidney disease were most common 2 to 6 months post transplant. Other complications, like acute kidney injury, hypertensive emergencies, hypertension, diabetes, dyslipidemia, and osteoporosis, were predominantly observed in the first month. Opportunistic infections peaked between 7 months and 1 year after transplantation. **Conclusions:** This study emphasizes the varied complications related to immunosuppressive therapy among different SOT recipients, delineating specific timeframes for each complication and maintenance regimen.

## 1. Introduction

Immunosuppressive therapies have a crucial role in enhancing graft survival and overall outcomes following organ transplantation [1,2]. The growing number of organ transplantations underscores the significance of post-transplantation care. According to the Korean Network for Organ Sharing (KONOS), there has been a steady increase in organ transplantations, with approximately 4000 transplants performed annually. Notably, from 2012 to 2021, organ transplantation witnessed a 46.5% rise. In 2021, the 5-year survival rate for transplants was reported at 86.16% [3].

Solid organ transplantation (SOT) relies on a range of immunosuppressive agents, including corticosteroids, anti-proliferative agents, calcineurin inhibitors (CNIs), mammalian target of rapamycin inhibitors (mTORi), polyclonal anti-lymphocyte antibodies, and monoclonal antibodies [4,5]. These immunosuppressive therapies have significantly reduced acute rejections, thereby increasing patient survival rates [6].

However, it is crucial to acknowledge the potential adverse events associated with prolonged use of immunosuppressive agents in transplant recipients. The use of these drugs can lead to significant complications, necessitating careful monitoring and management to strike a balance between preventing acute rejection and minimizing harmful effects [7]. These complications may include elevated blood pressure, hyperglycemia, hypercholesterolemia, infections, acute kidney disease, and neurological toxicities. Over time, these complications can progress to conditions such as diabetes mellitus, hypertension, dyslipidemia, osteoporosis, and chronic kidney disease [8,9,10,11,12]. Prolonged suppression of immune defenses resulting from immunosuppressive therapy can also lead to malignancies [8]. The immunosuppressant therapy used in transplantation has multiple implications, influencing graft survival, the patient’s prognosis after transplantation due to complications, and imposing a high burden of medical costs. Thus, there is a need for strategies to minimize and prevent the complications associated with prolonged immunosuppression post transplantation.

While prior research has often focused on individual complications or specific to particular organ transplantations, there has been a notable gap in comprehensive studies utilizing real-world data to provide a descriptive evaluation [8,9,11,12,13,14,15,16,17]. In this study, we aimed to comprehensively estimate the incidence and timing of complications associated with immunosuppressive therapy across various types of SOTs, employing the national claims database.

## 2. Materials and Methods

### 2.1. Data Source and Study Population

We conducted a comprehensive retrospective cohort analysis that included adult solid organ transplant recipients from the National Health Insurance Sharing Service (NHISS) claims database between 1 January 2007, and 31 December 2018. Extensive data were gathered, including demographic information (such as sex and age), as well as diagnoses, procedure types, and prescribed medications. The NHISS is a mandatory universal health coverage system that covers more than 97% of the population in South Korea, and the database contains all claims data for the entire national population of approximately 51 million people [18]. The initial cohort was established with patients who have used at least one immunosuppressant for transplantation (Supplementary Table S1). This study was conducted in accordance with the Strengthening the Reporting of Observational Studies in Epidemiology (STROBE) guidelines to ensure transparency and rigor in reporting. A completed STROBE checklist detailing compliance with these guidelines is provided in the Supplementary Materials. During the preparation of this manuscript, the author(s) used ChatGPT (version 4) for the purposes of editing and refining the content. The authors have reviewed and edited the output and take full responsibility for the content of this publication.

The study population comprised patients aged 18 years or older who had undergone transplantation of major organs, including the liver, kidney, heart, lung, and pancreas. Patients undergoing transplantation were identified using three primary insurance codes: The Z diagnostic code (Z940, Z941, Z942, and Z944) from the International Classification of Diseases, 10th Revision (ICD-10), the procedure code (Q804, Q805, Q806, Q808, Q8101, Q8102, Q814, Q815, and R328), and the V code (V005, V013, V014, V015, V081, V082, V083, V084, V085, V086, and V088) in the Rare Intractable Diseases (RID) database (Supplementary Table S2). The RID code pertains to the systematic reimbursement of co-payments for severe, chronic, or incurable diseases, thus reducing the out-of-pocket burden for patients [19].

We excluded patients with any record of organ transplantation in or before 2006 to ensure the inclusion of only first-time transplant recipients. In addition, we explicitly excluded liver re-transplantation cases by identifying procedure codes corresponding to liver re-transplants (Q814 and Q815). However, multi-organ transplantation and re-transplantation involving other organs during the study period (2007–2018) were not excluded by design and may have been included in the cohort.

Patients who had not received high-dose intravenous steroids with CNIs or other induction therapy were also excluded, as these individuals were presumed to have undergone transplantation outside of South Korea. Among the patients with a transplantation code, the index date was defined as the transplantation date and the initiation of induction therapy (basiliximab, anti-thymocyte globulin rabbit, or the first high-dose intravenous steroids with CNIs). We considered the use of immunosuppressant agents discontinued if there was a gap of more than 30 days between the last day of supply and the subsequent prescription. The observation period for complications began on the index date and continued until the earliest of the following events: 30 days after the discontinuation of immunosuppressive therapy, 3 years after the index date, the end of the study period (31 December 2018), or occurrence of death.

### 2.2. Ethics Approval and Consent to Participate

This study was approved by the Seoul National University Institutional Review Board (IRB No. E2112/004-002). The need for informed consent was waived by the Seoul National University Review Board, as only de-identified information was provided with no linkable data elements. All methods were carried out in accordance with the Declaration of Helsinki.

### 2.3. Outcome Definitions

In this study, the primary outcome was the incidence of complications related to immunosuppressive agents among recipients of SOTs. Our study focused on relatively short to mid-term complications following transplantation. Consequently, long-term outcomes such as death, cancer, and cardiovascular disease were not included in the analysis. Complications associated with immunosuppressive agents included infection, opportunistic infection, acute kidney injury (AKI), hypertensive emergency, chronic kidney disease (CKD), hypertension, diabetes, dyslipidemia, and osteoporosis. These were identified using ICD-10 diagnostic codes, supplemented by information on medication usage defined with ATC (Anatomical Therapeutic Chemical Classification) code, and records of hospitalization or visits to the emergency department (ED) (Supplementary Table S3). To account for newly occurring outcomes, a one-year baseline period was established. We have defined CKD, hypertension, diabetes mellitus, dyslipidemia, and osteoporosis as noncommunicable chronic diseases for further analysis.

We recognized the potential for episodes of serious infection, opportunistic infection, AKI, and hypertensive emergencies to occur more than once. If the same episode recurred within a 30-day window, it was considered a continuation of the previous outcome. Serious infection episodes were defined based on the primary diagnostic code, along with the administration of intravenous antibiotics (ATC code J) and records of hospitalization or ED visits. For opportunistic infections, tuberculosis was identified using its corresponding diagnostic codes with the prescription of tuberculosis-related antibiotics (ATC code J04A). Similarly, herpes zoster was identified using corresponding diagnostic codes and prescriptions for herpes zoster-related antiviral medications (acyclovir, famciclovir, and valaciclovir). All opportunistic infections, other than tuberculosis (mycobacterial infections) and herpes virus, were categorized as ‘other opportunistic infection’. Hypertensive emergencies were characterized by patients who received intravenous antihypertensive medications (esmolol, labetalol, and nicardipine) during hospitalizations or ED visits. AKI was identified by patients who were diagnosed with AKI and had corresponding hospitalization or ED visit records.

For noncommunicable chronic diseases, new-onset hypertension were determined by the initiation of new therapeutic agents alongside diagnostic codes in patients who did not have these conditions during the baseline period. New-onset diabetes was determined by the initiation of new therapeutic agents alone in patients who did not receive these agents during the baseline period. To minimize the impact of acute hyperglycemia, rapid-acting and short-acting insulins were excluded from the evaluation of diabetes mellitus. Additionally, for hypertension and diabetes mellitus, disease worsening was identified as the initial introduction of supplementary therapeutic agents during the follow-up period in cases where patients had previously undergone treatment for the same condition before transplantation. Dyslipidemia was detected by the initiation of newly prescribed lipid-lowering medications, with no anti-dyslipidemic drugs administered during the baseline period. Chronic kidney disease and osteoporosis were defined by the first presence of corresponding ICD-10 codes without evidence during the baseline period. These noncommunicable diseases included only the first occurrence after transplantation, and their persistence was not ascertained. The worsening of chronic diseases, such as hypertension and diabetes, was defined based on therapeutic escalation in accordance with clinical guidelines. For hypertension, worsening was identified when an additional antihypertensive medication was prescribed, indicating inadequate blood-pressure control despite prior treatment. Similarly, for diabetes, therapeutic intensification—such as the addition of a new antidiabetic agent or the initiation of insulin therapy—was used as a marker of disease progression. We acknowledge that some treatment modifications may result from adverse effects rather than true disease worsening. However, adverse drug reactions are typically managed by switching medications rather than adding supplementary agents. Therefore, by focusing on therapeutic intensification rather than medication changes due to side effects, we aimed to capture true disease progression while minimizing misclassification.

The analysis of maintenance immunosuppressive regimens focused on identifying the primary agents administered for the longest duration within the first three months post transplant. For each patient, the regimen with the highest cumulative exposure during this period was classified as their primary maintenance regimen. To examine potential shifts in regimens and their impact, we categorized complications based on the predominant regimens used at two key intervals: within the first 30 days and by 90 days post transplant. This approach allowed us to capture early and evolving patterns of immunosuppressant use and their association with complications. Since patients remain at risk for various complications over time, they were not censored after experiencing an initial complication.

### 2.4. Statistical Analysis

Continuous variables were presented as percentages, means, and standard deviations, with group comparisons conducted using *t*-tests.

Baseline characteristics of patients with and without complications were compared using *t*-tests for continuous variables. For categorical variables (e.g., age groups, CCI scores), a single Chi-square test was applied to assess overall differences across all levels of each variable, rather than conducting separate tests for each subgroup. This approach minimized the risk of Type I error due to multiple testing. These comparisons were exploratory, intended to describe the study population rather than test specific hypotheses.

All analyses for this study were conducted on transplant cases within the entire transplant population. To calculate the incidence rate of the outcomes, we divided the total number of observed outcomes by the total person years of immunosuppressant use. Total person years of immunosuppressant use was used as the denominator to account for the varying durations of exposure to these agents among patients. This approach provides a standardized measure of the incidence rate by relating the number of observed complications to the cumulative time patients were at risk while receiving immunosuppressive therapy. It ensures an evaluation of rates across different patient groups and transplant types, regardless of differences in follow-up periods. The incidence rates between transplant groups were intended to be descriptive and provide an overview of observed trends. For assessing the cumulative incidence of hypertension, diabetes, and dyslipidemia, we utilized Kaplan–Meier analysis. Results were considered statistically significant if the *p*-value was less than 0.05. SAS software version 9.4 (SAS Institute Inc., Cary, NC, USA) was used for all statistical analyses.

## 3. Results

### 3.1. Study Population

**Figure 1:**
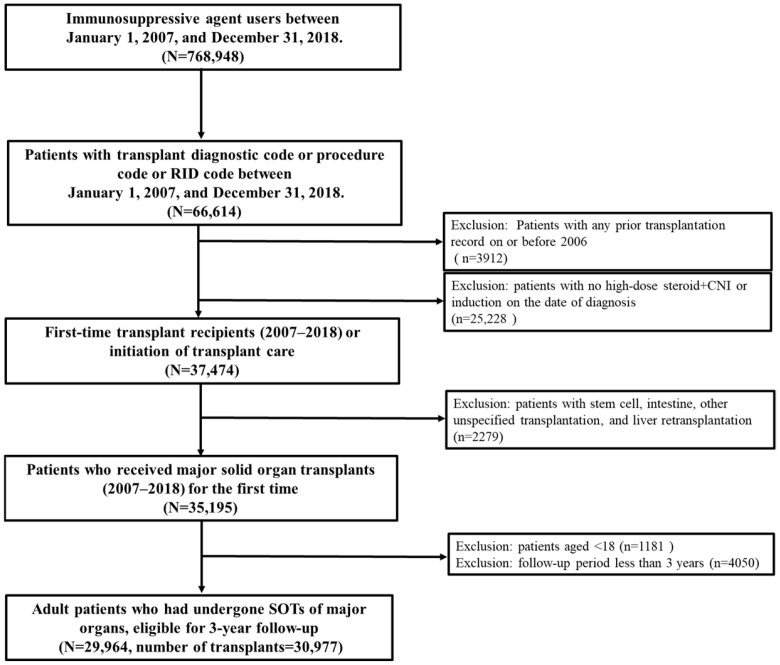
A flow chart of the patient selection process. Note: The final cohort consists exclusively of first-time transplant recipients, as patients with any transplantation history on or before 2006 were excluded using claims data. However, multi-organ and re-transplantation cases during the study period (2007–2018) were not excluded, except for liver re-transplantation, which was explicitly removed based on procedure codes.

From the NHISS national claims database, a total of 768,948 users of immunosuppressive agents were identified between 1 January 2007 and 31 December 2018. Among 62,702 patients with a transplantation code, we have excluded individuals who did not receive high-dose steroids and CNI, or induction therapy, on the date of diagnosis. A total of 37,474 patients with new transplantations or those who initiated transplant management in South Korea were identified. Among these, we identified 29,964 adult patients and 30,997 cases of major SOTs performed in South Korea from 2007 to 2018 that were eligible for a full 3-year follow up. These individuals comprised our final cohort (Figure 1).

Table 1 presents the demographic and clinical characteristics of all participants, distinguishing between transplant recipients who experienced at least one complication and those who did not during the study period. Out of the total 30,977 transplant cases, 19,681 (63.5%) were male, with a mean age at the time of transplantation of 50.03 ± 11.08 years. The largest proportion of patients (47.6%) fell into 50–64 age group, followed by those aged 35–49 years (34.3%), 18–34 years (12.2%), and those over 65 years of age (5.9%). Among all the transplantation cases, the majority were kidney transplants, accounting for 18,401 cases (59.4%). This was followed by liver transplants with 10,870 cases (35.1%), heart transplants with 957 cases (3.1%), pancreas transplants with 481 cases (1.6%), and lung transplants with 259 cases (0.8%).

Regarding induction therapy, 88.8% received basiliximab, while 11.2% received rabbit anti-thymocyte globulin (ATG). For the maintenance regimen, other than corticosteroids, tacrolimus (Tac) and mycophenolic acid (MPA) were used in 17,952 cases (58.0%), followed by Tac in 4846 cases (15.6%), cyclosporine (CsA) with MPA in 2149 cases (6.8%), and MPA monotherapy in 397 cases (1.3%).

Out of the 29,964 patients in the cohort, 3083 (10.3%) died during the 3-year study period.

### 3.2. Incidence Rate of Immunosuppressant-Related Complications by Organ Types

Table 2 presents the incidence rates of immunosuppressant-related complications among transplant recipients by the type of SOT. The overall incidence rate of complications was highest among lung transplant recipients (823.9/1000 patient years, 95% confidence interval [CI] 766.2–895.8), followed by heart transplant recipients (623.0/1000 patient years, 95% CI 592.2–649.5), kidney transplant recipients (539.0/1000 patient years, 95% CI 533.0–545.1), liver transplant recipients (504.9/1000 patient years, 95% CI 497.5–512.4) and pancreas transplant recipients (225.9/1000 patient years, 95% CI 211.1–241.4).

Lung transplant recipients were more likely to experience specific complications, with higher incident rates of serious infection (91.7/1000 patient years, 95% CI 71.6–115.7), opportunistic infection (278.9/1000 patient years, 95% CI 243–318.7), hypertensive emergency (47.8/1000 patient years, 95% CI 33.6–65.9), CKD (37.5/1000 patient years, 95% CI 25.1–53.8), and osteoporosis (60.7/1000 patient years, 95% CI 44.6–80.7). Among serious infections, pulmonary infection had the highest incidence rate among all subtypes (59.4/1000 patient years, 95% CI 43.5–79.2).

In contrast, kidney transplant recipients had a higher incidence of AKI (8.2/1000 patient years, 95% CI 7.4–8.9), urinary tract infections (18.8/1000 patient years, 95% CI 17.7–20.2) and hypertension (151.9/1000 patient years, 95% CI 148.7–155.2). Heart transplant recipients were more likely to develop dyslipidemia (84.7/1000 patient years), while liver transplant recipients were more likely to develop diabetes (116.8/1000 patient years, 95% CI 113.2–120.4). Pancreatic transplant recipients exhibited a higher risk of musculoskeletal infections (3.4/1000 patient years), when compared to recipients of other types of transplantation (Table 2).

### 3.3. Onset Timing of Immunosuppressant-Related Complications

Table 3 shows the incidence rates of complications related to immunosuppressant use in transplant recipients, stratified by time periods. The overall serious infection episode rate reached its highest point between 2 and 6 months, with the exception of GI infections and pulmonary infections. GI infections exhibited their peak incidence rates within the initial one-month period, while pulmonary infections peaked between seven months and one year after transplantation. Opportunistic infections, notably tuberculosis and herpes, were more frequently observed after the first 2 months post transplantation, with their peak incidence occurring between the 7-month and 1-year period. Among other complications, AKI, hypertensive emergency, hypertension, diabetes mellitus, dyslipidemia, and osteoporosis were most frequently observed during the initial one month of immunosuppressive therapy, while CKD cases occurred during the 2- to 6-month period (Table 3). Importantly, all complications except opportunistic infections occurred within six months from the initiation of immunosuppressive therapy. It is important to note that complications identified within the first 30 days post transplant may partly reflect post-surgical complications, in addition to those directly associated with immunosuppressive therapy. This distinction should be considered when interpreting early complication trends.

### 3.4. Cumulative Incidence of Noncommunicable Chronic Complications

**Figure 2:**
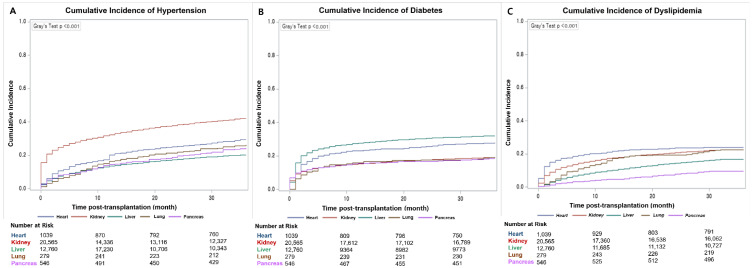
Cumulative incidence of hypertension (**A**), diabetes mellitus (**B**) and dyslipidemia (**C**) during the follow-up period after major SOTs.

Among noncommunicable chronic diseases, hypertension, dyslipidemia, and diabetes mellitus were the three most frequently encountered complications. When considering the cumulative 1-year incidence of these three conditions, kidney transplant exhibited the highest cumulative incidence rate of 0.32 (95% CI, 0.32–0.33) for hypertension, and liver transplant had the highest cumulative incidence rate of 0.28 (95% CI, 0.17–0.28) for diabetes. Heart transplant demonstrated the highest cumulative incidence of 0.22 (95% CI, 0.19–0.24) for dyslipidemia (Figure 2).

### 3.5. Incidence of Complications by Maintenance Regimen

Regarding the maintenance regimen of immunosuppressive therapy within the first 90 days post transplant, the combination of Tac with MPA emerged as the most prevalent maintenance regimen across all types of SOTs. For kidney transplants, the primary maintenance therapy consisted of a combination of Tac and MPA, followed by a combination of CsA and MPA (Supplementary Table S4). An examination of maintenance regimen patterns during the three years post transplant revealed an increase in the utilization of mTORi in heart transplantation by the third month. Generally, patients maintained the same therapy during the first year post transplant. Subsequently, there was a decline in combination therapy after the first year post transplant, particularly in liver and heart transplantation (Supplementary Figure S1).

**Figure 3:**
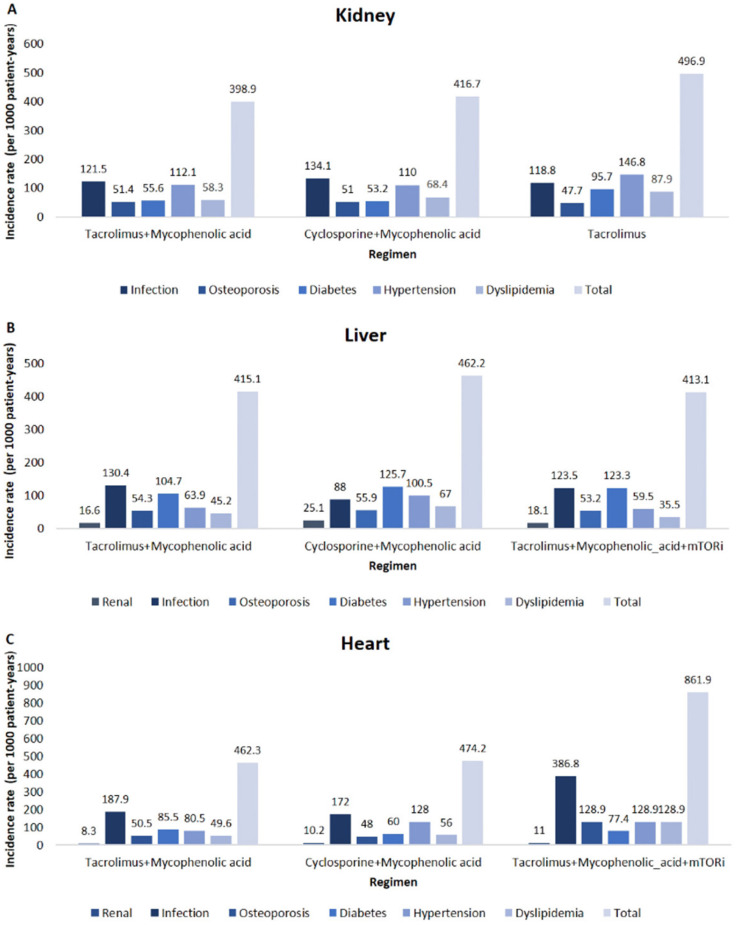
Overall incidence of complications by regimen in kidney (**A**), liver (**B**), and heart (**C**) transplant recipients.

The incidence of complications associated with different immunosuppressive regimens was observed within our study population, categorized by the major regimen used during the initial 30 days in each organ type. We estimated the incidence rate based on major maintenance therapy used within 30 days post transplantation. Regimens that included CsA in liver and heart transplantation demonstrated a higher incidence of renal dysfunction compared to other complications. Conversely, regimens incorporating MPA in kidney, liver, and pancreatic transplantation demonstrated a higher incidence of infection compared to other regimens. In heart transplant cases, the use of mTORi was associated with a higher incidence of all complications. When comparing complications between the Tac + MPA and CsA + MPA regimens, it was evident that the combination therapy containing CsA had a generally higher occurrence of complications in kidney liver and heart transplantation (Supplementary Table S5). The summary of overall incidence of complications by regimen in kidney (A), liver (B) and heart (C) transplant recipients are displayed in Figure 3.

For kidney transplantation, the Tac + MPA regimen had a relatively high incidence of infections, with 496.6 per 1000 patient years. However, this regimen demonstrated a lower incidence of diabetes compared to Tac alone. The CsA + MPA regimen exhibited the highest infection rate, at 134.1 per 1000 patient years. Despite this high infection rate, it was associated with a lower incidence of diabetes compared to Tac alone. In contrast, Tac alone had the highest incidence of diabetes, at 95.7 per 1000 patient years.

In liver transplantation, the CsA + MPA regimen had the highest incidence of complications, with 462.2 per 1000 patient years compared to other regimens. Specifically, CsA + MPA was associated with a higher incidence of diabetes compared to the Tac + MPA regimen (125.7 per 1000 patient years vs. 104.7 per 1000 patient years). Additionally, dyslipidemia was more frequently observed in the CsA + MPA group, with an incidence of 67 per 1000 patient years, compared to 45.2 per 1000 patient years in the Tac + MPA group.

For heart transplantation, the Tac + MPA + mTORi regimen had the highest total complication rate, with an incidence of 861.9 per 1000 patient years. Infection rates were higher in the Tac + MPA group compared to the CsA + MPA group (187.9 per 1000 patient years vs. 172 per 1000 patient years), suggesting a greater infection risk in patients on Tac-based regimens. The incidence of diabetes was also higher in the Tac + MPA group compared to the CsA + MPA group (85.5 per 1000 patient years vs. 60 per 1000 patient years), indicating that Tac-based regimens may be more likely to induce new-onset diabetes in heart transplant recipients. However, hypertension was more common in the CsA + MPA group, with an incidence of 128 per 1000 patient years, compared to 80.5 per 1000 patient years in the Tac + MPA group, suggesting a higher risk of hypertension in patients receiving CsA-based regimens. To comprehend the shift in maintenance regimens over time, we also investigated the incidence of complications based on the major regimens during the initial 90 days (Table 4). We have presented the incidence of complications by major regimen used over 90 days in Table 4. The overall incidence of complications varied by organ type and immunosuppressive regimen. Lung and pancreas transplant recipients exhibited the highest overall complication rates, with Tac + MPA in lung recipients reaching 1293.6 per 1000 patient years, followed by Tac + MPA in pancreas recipients (757.1 per 1000 patient years). In contrast, kidney and liver transplant recipients generally experienced lower complication rates, ranging from 398.3 to 484.3 per 1000 patient-years. Among kidney transplant recipients, Tac + MPA (411.0 per 1000 patient years) had a slightly lower overall complication rate compared to CsA + MPA (443.2) and Tac alone (484.3). Infection rates were highest with CsA + MPA (147.2 per 1000 patient years), while diabetes rates were highest in Tac alone (71.3 per 1000 patient years). For liver transplant recipients, Tac alone was the lowest overall complication rate (398.3 per 1000 patient years), whereas CsA + MPA had the highest rate (467.4 per 1000 patient years). Infection rates were similar across regimens (134.1–147.2 per 1000 patient years), and diabetes incidence was comparable (54.7–59.9 per 1000 patient years). Heart transplant recipients on Tac + MPA + mTORi had the highest overall complication rate (698.6 per 1000 patient years) compared to Tac + MPA (468.5) and CsA + MPA (508.1). Notably, infection rates were highest in Tac + MPA + mTORi (237.4 per 1000 patient years), and this regimen was also associated with the highest rates of diabetes (110.4 per 1000 patient years) and dyslipidemia (160.1 per 1000 patient years). We observed that the outcomes of the primary regimen employed in the first 30 days and 90 days remained consistent.

## 4. Discussion

This study stands out as the first large-scale cohort analysis in South Korea to comprehensively assess complications associated with immunosuppressive agents in all major solid organ transplant (SOT) recipients. Our nationwide approach has provided a detailed understanding of these complications, revealing that lung transplant recipients experience the highest incidence, followed by heart, kidney, liver, and pancreas recipients. These insights may serve as a fundamental framework for making informed decisions regarding medication choices and improving post-transplant monitoring, thereby advancing patient care and enhancing patient safety. This study was designed to include only first-time transplant recipients. To achieve this, we excluded all patients with any record of transplantation in or before 2006 using claims data. As a result, the final cohort consists exclusively of patients who received a new transplant for the first time between 2007 and 2018. However, patients who underwent multi-organ transplantation or re-transplantation during the study period (from 2007 onward) were not excluded by the initial selection criteria. Given that early complication rates are known to be higher in these patient groups, their inclusion may have introduced confounding effects into our findings. Our findings indicate that the majority of complications, with the notable exception of opportunistic infections like tuberculosis and herpes which peak between the six-month and one-year mark, typically manifest within the first six months following the initiation of immunosuppression. This pattern underscores a critical period for intensified patient care and monitoring. While comparing our results with prior studies is challenging due to methodological differences, our findings on infection rates and specific patterns in different transplant types resonate with those from a similar 2020 Swiss cohort study [17], thereby reinforcing the validity of our conclusions.

In both our study and the Swiss research, lung transplant recipients exhibited the highest cumulative infection rates at one year post transplant, compared to other major SOT types. Furthermore, our study identified distinct infection patterns in various transplant types, echoing the findings of the Swiss study [17]. Notably, liver transplant recipients primarily faced infections in the hepatic and other intra-abdominal regions, while kidney and lung transplant recipients were more susceptible to urinary and respiratory tract infections, respectively.

An important observation in our study was the relatively high incidence of tuberculosis (TB), particularly among liver and lung transplant recipients. This is likely attributable to the greater immunosuppressive burden required for these organs, as well as Korea’s intermediate TB burden (50 per 100,000 population) [20]. Although the nature of our data does not allow us to distinguish between reactivation and de novo TB, the predominance of latent TB reactivation in endemic areas is well documented [21].

Our findings on opportunistic infections also align with global data. Lung transplant recipients exhibited the highest incidence, consistent with prior studies identifying them as the most vulnerable to opportunistic infections due to intensified immunosuppressive regimens and mucosal vulnerability [11]. Similar patterns were observed in Korean nationwide studies despite declining TB incidence [22].

These data emphasize the need for region-specific infection control measures, including enhanced TB screening and opportunistic infection prophylaxis, especially for recipients of high-risk organs.

In terms of specific complications, our study aligns with previous research on the prevalence of hypertension in kidney transplant recipients [23,24] and post-transplant diabetes mellitus (PTDM) in liver transplant patients [25,26,27]. Our findings further emphasize the need for rigorous hypertension management, especially in kidney transplant recipients, and alert clinicians to the high risk of PTDM post-liver transplantation.

The prevalence of dyslipidemia in heart transplant recipients, observed in our study, resonates with the existing literature [28], highlighting the impact of maintenance therapy with mTOR inhibitors on lipid profiles [29,30].

The consistency of our observations regarding the incidence rates of diabetes in patients receiving different immunosuppressive regimens with existing meta-analysis findings underscores the importance of regimen selection in managing post-transplant diabetes risk [31]. We acknowledge that previous studies have demonstrated a higher risk of diabetes with tacrolimus compared to cyclosporine. However, our findings did not consistently reflect this trend. While tacrolimus use was associated with higher diabetes incidence in kidney and heart transplant recipients, the opposite trend was observed in liver transplant recipients. This discrepancy may be explained by selection bias, as patients at elevated risk for diabetes may have been preferentially prescribed alternative immunosuppressant. As such, we cannot confidently establish a direct cause-and-effect relationship between tacrolimus and diabetes in our study. Future prospective research incorporating detailed clinical variables will be essential to clarify this association.

Additionally, when interpreting our findings related to kidney transplantation, it is essential to recognize that all kidney transplant recipients, by definition, have chronic kidney disease (CKD) as the underlying indication for transplantation. Unlike other chronic diseases such as diabetes or hypertension, where new-onset cases can be identified post-transplant, CKD is inherently present before transplantation. Therefore, our approach—relying on the first recorded ICD-10 codes without prior documentation during the baseline period—was not appropriate for assessing CKD incidence or progression post transplant. As a result, CKD was not included in our post-transplant outcomes analysis (Table 2). Post-transplant CKD progression may reflect chronic allograft dysfunction, which has significant implications for graft survival and patient outcomes. However, distinguishing chronic allograft dysfunction from baseline CKD using claims data poses methodological challenges due to limited granularity in clinical information, such as serial estimated glomerular filtration rate (eGFR) measurements or biopsy results. Our study did not specifically address chronic allograft dysfunction, and further research incorporating detailed clinical data would be necessary to investigate this important outcome.

In this study, we present an extensive and descriptive overview of complications across major solid organ transplants (SOTs), encompassing kidney, liver, heart, lung, and pancreas transplants. Leveraging a robust longitudinal dataset from a national claims database, our research uniquely chronicles the evolution of post-transplant complications over an extended follow-up period. We delve into the intricacies of how these complications manifest differently across organ types, providing a nuanced understanding of both common and organ-specific challenges. This comprehensive approach marks our study as the first large-scale cohort analysis in South Korea, including the entire national population and utilizing real-world data. By identifying variations in complication incidence rates among different organ transplant types, various immunosuppressive regimens, and across different time periods, our study not only contributes significantly to transplant medicine but also offers valuable insights for healthcare professionals. These insights may serve as a fundamental framework for making informed decisions regarding medication choices and improving post-transplant monitoring, thereby advancing patient care and enhancing patient safety.

While acknowledging the significance of our study, it is important to recognize its limitations. First, while ICD-10 codes are widely used and provide standardized classifications for diseases and conditions, there is an inherent risk of inaccuracies due to several factors. These include coding errors, where diagnoses may be incorrectly assigned or misclassified; incomplete documentation, where healthcare providers might fail to record all relevant diagnoses or complications; and variability in coding practices across different institutions. Additionally, there may have been instances where not all diagnostic codes could be accurately assigned, potentially leading to omissions or misclassifications of diagnoses. Second, while we recognize that death, cancer, and cardiovascular diseases are major contributors to long-term graft and patient attrition following transplantation, our study specifically focused on short- to mid-term complications occurring during the early post-transplant period. This period is characterized by the most intensive use of immunosuppressive regimens, during which complications are typically more frequent and directly related to the management of immunosuppression. We applied a fixed three-year follow-up period across all transplant recipients to ensure a consistent observation window and minimize potential bias from differences in follow-up duration. During this period, 3083 patients (10.3%) died. While some of these deaths may have been influenced by immunosuppression-related complications, early mortality can also result from pre-existing comorbidities, perioperative complications, or infections unrelated to immunosuppressive therapy. Given this complexity, our study focused on early post-transplant complications rather than overall mortality trends. Nonetheless, as early complications may contribute to later adverse outcomes, optimizing early post-transplant management remains crucial. Future research with extended follow up is needed to clarify the long-term impact of these early complications on patient and graft survival.

Third, era effects—temporal changes in clinical practices, treatment protocols, or medication use that occur over extended periods—should be considered when interpreting our research results. The shift in immunosuppressive regimens, particularly the transition from cyclosporine to tacrolimus, occurred between 2006 and 2007 in South Korea [32]. As our study period began after this transition had been largely completed, we believe that the era effects related to this shift have a limited impact on our findings. Consequently, our results likely reflect broader, stabilized trends in immunosuppressive therapy during this period. Nonetheless, we acknowledge that further analyses stratified by transplant year could provide additional insights into residual era effects and their potential influence on long-term outcomes.

In addition, the potential misclassification of chronic kidney disease (CKD) diagnoses in the early post-transplant period should be considered. According to KDIGO guidelines, CKD requires at least three months of persistent kidney dysfunction for diagnosis. However, because our dataset is based on claims data, CKD codes may reflect the timing of documentation rather than the precise onset of disease. Consequently, CKD diagnoses recorded within the first month post transplant may indicate pre-existing kidney dysfunction or early documentation rather than truly incident CKD. Due to data limitations, we were unable to conduct further analyses to differentiate these cases. Future research incorporating longitudinal clinical data will be necessary to validate the timing and accuracy of CKD diagnoses. Furthermore, our exclusive reliance on data from the national health insurance claims database limited our ability to incorporate factors such as lifestyle information, laboratory values, and specific details regarding donors’ characteristics such as ABO and HLA compatibilities. Also, our study was restricted to residents of South Korea, making it challenging to generalize the results to other countries or ethnic groups. Lastly, our study outcomes were established using an operational definition based on disease codes, medication codes, and administrative codes. However, recognizing the potential inaccuracies in the operational definition when using claims data, we expanded our definitions to include drug usage, hospitalization, or emergency room visits in addition to diagnostic codes to address these concerns. By employing standardized definitions that utilize ICD-10 codes, ATC codes, and administrative codes for the major complications related to immunosuppressant usage, our study offers a comprehensive and structured review of major complications, ensuring consistent and precise measurements.

This study is subject to limitations stemming from the nature of the original data sources. The absence of standardized and systematic data collection methods at the time of data acquisition has introduced potential biases, thereby constraining the generalizability and strength of the conclusions drawn. Specifically, the national claims database used in this study does not contain critical clinical variables such as laboratory values (e.g., serum creatinine, glucose levels), immunosuppressant drug trough levels, donor characteristics (e.g., HLA/ABO compatibility), or lifestyle factors (e.g., smoking status, BMI). In addition, the use of diagnostic and medication codes may result in the misclassification of outcomes, especially in distinguishing disease progression from adverse drug reactions or post-surgical complications.

To overcome these limitations, future research would benefit from the implementation of enhanced data management infrastructures, such as the establishment of a dedicated national transplant registry that systematically links claims data with electronic medical records (EMR). Such a registry should incorporate structured templates for capturing time-stamped laboratory results, drug levels, adverse event causality assessments, and longitudinal follow up. Integration with existing infrastructure such as the Korean Organ Transplant Registry (KOTRY) and the National Health Insurance System (NHIS) could facilitate nationwide data collection and monitoring. These improvements would enhance the validity of complication assessments, support real-time safety surveillance, and enable more robust risk-prediction models to guide post-transplant management strategies.

## 5. Conclusions

Our study provides a nuanced view of the landscape of immunosuppressive-related complications in SOT recipients, with particular emphasis on the early post-transplant period. These insights are invaluable for customizing monitoring and management strategies for SOT recipients, acknowledging the specific timelines for each complication. This comprehensive analysis not only advances our understanding of post-transplant care but also sets a foundation for future research aimed at improving patient outcomes in this critical area of medicine.

## Figures and Tables

**Table 1:** The baseline characteristics of the study population.

Variables	Transplant Recipients ^a^ (*N* = 30,977)	With Any Complications (*N* = 23,915)	Without Complication (*N* = 7062)	*p*-Value
Age (mean ± SD)	50.03 ± 11.08	50.48 ± 11.0	49.54 ± 11.15	<0.001
18–34	3777 (12.2)	2716 (11.0)	1061 (15.1)	<0.001
35–49	10,630 (34.3)	8002 (33.3)	2.628 (36.7)	
50–64	14,740 (47.6)	11,655 (49.2)	3085 (43.6)	
≥65	1830 (5.9)	1542 (6.5)	288 (4.6)	
Male, *n* (%)	19,681 (63.5)	14,154 (64.5)	5527 (61.2)	<0.001
Comorbid disease				<0.001
Hypertension	23,004 (22.4)	17,244 (23.1)	5760 (20.5)	<0.001
Dyslipidemia	20,332 (19.8)	14,937 (20.0)	5395 (19.2)	<0.001
Liver disease	19,077 (18.5)	13,043 (17.4)	6034 (21.5)	<0.001
Diabetes mellitus	18,608 (18.1)	13,252 (17.7)	5356 (19.0)	<0.001
Heart Failure and cardiomyopathy	18,618 (18.1)	13,828 (18.5)	4790 (17.0)	<0.001
Coronary artery disease	3251 (3.2)	2460 (3.3)	791 (2.8)	<0.001
CCI score (mean ± SD)	6.11 ± 2.48	6.56 ± 2.34	6.30 ± 2.31	<0.001
0–2	752 (2.4)	466 (2.1)	286 (3.2)	<0.001
3–4	5216 (16.8)	3300 (15.0)	1916 (21.2)	
5–6	10,165 (32.8)	7111 (32.4)	3054 (33.8)	
>6	14,844 (47.9)	11,072 (50.4)	3772 (41.8)	
Organ				<0.001
Kidney	18,401 (59.4)	13,663 (62.2)	4738 (52.5)	<0.001
Liver	10,870 (35.1)	7053 (32.1)	3817 (42.3)	<0.001
Heart	957 (3.1)	709 (3.2)	248 (2.7)	0.0255
Pancreas	481 (1.6)	314 (1.4)	167 (1.8)	0.0067
Lung	259 (0.8)	203 (0.9)	56 (0.6)	0.0075
Retransplantation ^b^	98 (0.3)	74 (0.3)	24 (0.3)	0.2918
Multi-organ transplantation ^c^	2370 (7.7)	2010 (8.4)	360 (5.1)	<0.001
Kidney-liver	1063 (44.9)	904 (45.0)	159 (44.2)	<0.001
Kidney-pancreas	809 (34.1)	685 (34.1)	124 (34.4)	<0.001
Liver-pancreas	490 (20.7)	413 (20.5)	77 (21.4)	<0.001
Heart-lung	8 (0.3)	8 (0.4)	0 (0.0)	0.1299
Induction regimen	27,792 (89.7)	22,132 (92.5)	6563 (92.9)	<0.001
Basiliximab	24,683 (88.8)	19,020 (85.9)	5962 (90.8)	
ATG rabbit	3109 (11.2)	3112 (14.1)	601 (9.2)	
Maintenance regimen other than steroids				<0.001
Tac + MPA	17,952 (58.0)	13,695 (57.3)	4257 (60.3)	
Tacrolimus	4846 (15.6)	3559 (14.9)	1287 (18.2)	
CsA + MPA	2149 (6.9)	1800 (7.5)	349 (4.9)	
Cyclosporine	804 (2.6)	639 (2.7)	165 (2.3)	
Mycophenolate	397 (1.3)	301(1.3)	96(1.4)	
Others	4829 (15.6)	3825 (16.4)	908 (12.8)	
Duration of steroid therapy				<0.001
≤30 days	7319 (23.6)	5009 (20.9)	2310 (32.7)	
30–90 days	9431 (30.4)	7378 (30.9)	2053 (29.1)	
>90 days	14,227 (46.1)	11,528 (48.2)	2699 (38.2)	

CCI, Charlson comorbidity index; Tac, tacrolimus; CsA, cyclosporine. ^a^ Patients with any transplantation record in or before 2006 were excluded to ensure inclusion of first-time transplant recipients only. ^b^ Liver re-transplantation cases were explicitly excluded using procedure code–based filtering. However, re-transplantation involving other organs (e.g., kidney, heart) that occurred from 2007 onward may have been included, as these cases were not excluded by design. Therefore, the re-transplantation row in this table reflects only those captured during the study period (2007–2018), excluding liver cases. ^c^ Multi-organ and non-liver re-transplantation cases occurring during the study period (2007–2018) were not excluded at cohort entry but are not presented in this baseline demographic table.

**Table 2:** Incidence rates of immunosuppressant complications in transplant patients by organ types.

Complications	Incidence Rate Per 1000 Patient Years (95% CI)					
	All	Kidney	Liver	Heart	Lung	Pancreas
Overall	504.1 (499.7–508.6)	539.0 (533.0–545.1)	504.9 (497.5–512.4)	623.0 (592.2–649.5)	823.9 (766.2–895.8)	225.9 (211.1–241.4)
Serious infection	43.7 (42.5–45.1)	49.7 (47.8–51.5)	34.2 (32.3–36.2)	57.8 (49.5–67.2)	91.7 (71.6–115.7)	22.1 (17.6–27.3)
Pulmonary infection	11.0 (1.4–11.7)	11.0 (10.2–11.9)	9.5 (8.5–10.6)	22.4 (17.4–28.6)	59.4 (43.5–79.2)	5.2 (3.2–8.0)
GI infection	98.1 (11.9–13.3)	5.5 (4.9–6.1)	14.0 (12.8–15.3)	13.6 (9.7–18.5)	15.5 (8.0–27.1)	2.1 (0.9–4.1)
Urinary tract infection	12.6 (11.9–13.3)	18.8 (17.7–20.2)	3.8 (3.2–4.5)	6.5 (3.9–10.1)	6.5 (2.1–15.1)	6.0 (3.8–9.0)
Skin infection	5.3 (4.9–5.8)	6.1 (5.5–6.8)	3.7 (3.1–4.4)	9.9 (6.6–14.2)	5.2 (1.4–13.2)	4.4 (2.6–7.1)
ENT infection	3.7 (3.4–4.1)	4.2 (3.7–4.7)	3.3 (2.7–3.9)	3.7 (1.9–6.7)	5.2 (1.4–13.2)	0.8 (0.2–2.3)
Musculoskeletal infection	1.9 (1.7–2.2)	2.3 (1.9–2.7)	1.3 (0.9–1.7)	2.0 (0.7–4.4)	0.0	3.4 (1.8–5.8)
Other infection	12.1 (11.4–12.8)	13.5 (12.6–14.5)	10.0 (9.0–11.1)	17.3 (12.9–22.8)	23.2 (13.8–36.7)	4.7 (2.8–7.4)
Opportunistic infection	113.3 (11.2–115.4)	105.8 (103.1–108.5)	126.0 (122.3–129.8)	152.0 (138.3–166.8)	278.9 (243–318.7)	47.8 (41.1–55.2)
Tuberculosis	29.9 (28.8–30.9)	21.7 (20.5–22.9)	44.8 (42.6–47.0)	24.5 (19.2–30.8)	52.9 (38.0–71.8)	15.3 (11.7–19.8)
Herpes	73.3 (72.1–75.5)	72.1 (69.9–74.3)	76.6 (73.8–79.6)	106.1 (94.7–118.6)	189.8 (160.4–223.1)	27.5 (22.5–33.3)
Other opportunistic infection	9.6 (9.0–10.3)	12.0 (11.2–13.0)	4.6 (3.9–5.4)	21.4 (16.5–27.4)	36.2 (24.0–52.3)	4.9 (3.0–7.7)
Acute kidney injury	6.2 (5.7–6.7)	8.2 (7.4–8.9)	3.6 (3.0–4.3)	2.7 (1.2–5.4)	7.7 (2.8–16.9)	2.3 (1.1–4.4)
Hypertensive emergency	20.0 (19.1–20.9)	22.0 (20.8–23.2)	25.0 (23.4–26.7)	21.4 (16.5–27.4)	47.8 (33.6–65.9)	29.6 (24.4–35.6)
Chronic kidney disease	20.1 (18.6–21.7)	-	14.7 (13.4–16.0)	16.0 (11.7–21.3)	37.5 (25.1–53.8)	6.5 (4.2–9.6)
Hypertension	118.0 (115.9–120.2)	151.9 (148.7–155.2)	73.9 (71.1–76.8)	104.1 (92.7–116.4)	94.3 (73.9–118.5)	34.3 (28.7–40.6)
Diabetes mellitus	82.8 (81.0–84.6)	68.9 (66.8–71.1)	116.8 (113.2–120.4)	99.0 (87.9–111.0)	69.7 (52.4–91.0)	26.2 (21.4–31.9)
Dyslipidemia	105.7 (103.1–108.4)	81.4 (79.0–83.7)	61.1 (58.5–63.7)	84.7 (74.5–95.9)	81.4 (62.5–104.1)	13.5 (10.1–17.7)
Osteoporosis	6.1 (5.5–6.7)	9.6 (8.8–10.5)	10.7 (9.6–11.8)	9.5 (6.3–13.8)	60.7 (44.6–80.7)	3.1 (1.6–5.4)

GI, gastrointestinal; ENT, ear/nose/throat.

**Table 3:** Incidence rates of immunosuppressant-related complications of transplant patients by timing of occurrence.

Complications	Complication Episodes (*N*)	Incidence Rate Per 1000 Patient Year (95% CI)			
		0–1 Month	2–6 Month	7 Month–1 Year	2–3 Year
Overall	41,332	662.8 (631.4–695.0)	606.9 (593.4–620.6)	394.1 (384.3–404.0)	263.2 (259.7–267.9)
Serious infection	4368	76.8 (66.5–88.4)	124.5 (118.5–130.8)	82.4 (77.9–87.0)	58.1 (56.2–60.0)
GI infection	863	21.2 (15.9–27.6)	18.0 (15.7–20.5)	9.2 (7.7–10.8)	7.3 (6.6–8.0)
ENT infection	372	0.8 (0.1–2.8)	3.7 (2.7–4.9)	4.7 (3.7–5.9)	4.1 (3.6–4.6)
Pulmonary infection	1099	4.7 (2.4–8.2)	18.3 (16.0–20.8)	17.4 (15.4–19.6)	14.0 (13.1–15.0)
Urinary tract infection	1258	10.6 (7.0–15.4)	28.0 (25.2–31.1)	20.5 (18.3–22.9)	17.0 (15.9–18.0)
Musculoskeletal infection	193	0.0	1.4 (0.8–2.2)	2.0 (1.4–2.9)	1.7 (1.4–2.1)
Skin infection	529	5.5 (3.0–9.2)	10.0 (8.3–11.9)	6.1 (4.9–7.4)	4.2 (3.7–4.8)
Other infection	1209	23.9 (18.3–30.7)	32.3 (29.2–35.5)	13.4 (11.6–15.3)	5.9 (5.3–6.6)
Opportunistic infection	11,313	74.5 (64.3–85.9)	178.8 (171.5–186.3)	182.8 (176.2–189.6)	113.9 (111.2–116.6)
Tuberculosis	2981	18.8 (13.9–25.0)	48.2 (44.5–52.2)	74.8 (70.6–79.3)	31.6 (30.2–33.0)
Herpes	7371	27.8 (21.7–35.1)	85.5 (80.5–90.7)	96.0 (91.2–100.9)	78.1 (75.9–80.4)
Other opportunistic infection	961	27.8 (21.7–35.1)	45.0 (41.5–48.9)	12.0 (10.3–13.8)	4.2 (3.7–4.7)
Acute kidney injury	618	24.3 (18.6–31.2)	16.2 (14.0–18.5)	7.3 (6.1–8.8)	4.0 (3.5–4.5)
Hypertensive emergency	1998	261.9 (242.4–282.5)	44.4 (40.8–48.3)	20.4 (18.2–22.7)	16.0 (15.0–17.1)
Chronic kidney disease	621	52.8 (28.9–88.6)	124.1 (105.8–144.6)	64.5 (52.7–78.1)	53.4 (47.9–59.4)
Hypertension	11,788	142.5 (137.7–147.4)	27.3 (26.3–28.2)	11.5 (10.9–12.1)	6.7 (6.5–6.9)
Diabetes mellitus	2334	101.7 (96.6–106.9)	54.2 (52.5–55.9)	10.6 (10.0–11.3)	5.1 (4.9–5.4)
Dyslipidemia	7159	43.6 (40.3–47.1)	38.1 (36.8–39.6)	14.8 (14.0–15.6)	6.7 (6.4–7.0)
Osteoporosis	1013	2.6 (1.9–3.5)	1.6 (1.3–1.9)	1.1 (0.9–1.3)	0.9 (0.8–1.0)

GI, gastrointestinal; ENT, ear/nose/throat.

**Table 4:** Incidence of complications by major regimen used over 90 days.

Organ	Regimen	Exposure	Total	Renal ^a^	Infection ^b^	Osteoporosis	Diabetes	Hypertension	Dyslipidemia
		(Patient-Years)	Per 1000 Patient Years						
Kidney	Tac + MPA	47,041	411.0	-	127.9	51.1	52.2	105.1	55.8
	CsA + MPA	7634	443.2	-	147.2	50.6	50.4	104	66.1
	Tac	4879	484.3	-	146.5	51.9	71.3	121.7	65.4
Liver	Tac + MPA	20,875	406.4	16.0	134.1	54.7	94.3	61.4	45.9
	Tac	8143	398.3	17.4	138.2	55.9	92.2	58	36.6
	CsA + MPA	7634	467.4	19.1	147.2	59.9	93.7	87.6	59.9
Heart	Tac + MPA	2290	468.5	12.2	196.9	49.8	83.4	78.2	48.0
	CsA + MPA	295	508.1	20.3	220.2	44.0	57.6	108.4	57.6
	Tac + MPA + mTORi	181	698.6	16.6	237.4	60.7	110.4	160.1	110.4
Pancreas	Tac + MPA	1392	757.1	236	137.9	57.5	34.4	58.9	23.0
	Tac	80	275.0	50.0	62.5	62.5	0.0	87.5	12.5
Lung	Tac + MPA	356	1293.6	42.2	236.2	115.3	73.1	64.7	61.9

^a^ Renal dysfunction was defined by composite of chronic kidney disease and acute kidney injury. ^b^ Infection was defined by composite of serious infection and opportunistic infection. Tac, tacrolimus; MPA, mycophenolic acid; CsA, cyclosporine; mTORi, mammalian target of rapamycin inhibitor.

## Data Availability

The datasets used in the study can be accessed from the Health Insurance Review and Assessment service, but their use is limited due to licensing and not intended for public release. However, data will be shared on reasonable request to the corresponding author with the permission of the Health Insurance Review and Assessment service.

## Supplementary Materials

The following supporting information can be downloaded at: https://www.mdpi.com/article/10.3390/jcm14103602/s1, Figure S1: A, Immunosuppressant pattern of use for maintenance regimen in liver transplant recipients. B, Kidney transplant recipients. C, Heart transplant recipients; Table S1: ATC codes for immunosuppressants used in transplant recipients; Table S2: Diagnostic, procedure, and rare intractable disease codes for transplantation; Table S3: Category of immunosuppressant-related harm according to disease code (ICD-10 code) and treatment medication (WHO-ATC code); Table S4: Frequency of immunosuppressant regimen by organ types; Table S5: Incidence rate of complications by major regimen used over 30 days.

## Abbreviations

AKI (acute kidney injury); ATC (Anatomical Therapeutic Chemical Classification), ATG (anti-thymocyte globulin); CI (confidence interval); CKD (chronic kidney disease), CNIs (calcineurin inhibitors); CsA (cyclosporine); ED (emergency department); eGFR (estimated glomerular filtration rate); ENT (ear/nose/throat); GI (gastrointestinal); HCV (hepatitis C virus); ICD-10 (International Classification of Diseases, 10th Revision); KNOS (Korean Network for Organ Sharing); MPA (mycophenolic acid); mTORi (mammalian target of rapamycin inhibitor); NASH (nonalcoholic steatohepatitis); NHISS (National Health Insurance Sharing Service); PTDM (post-transplant diabetes mellitus); RID (Rare Intractable Diseases); SOT (solid organ trans-plant); Tac (tacrolimus).
