# Supplementary material for: Complications Associated with Immunosuppressive Agents in Solid Organ Transplant Recipients: A Nationwide Analysis
Source: J Clin Med. 2025 May 21;14(10):3602. doi: 10.3390/jcm14103602 (PMC12112735; doi:10.3390/jcm14103602)
Supplement: Supplementary file 1 [file jcm-14-03602-s001.zip › jcm-3619809-supplementary.pdf]

Article Title: Complications Associated with Immunosuppressive Agents in Solid Organ Transplant Recipients: A Nationwide Analysis

Supplementary Table S1. ATC codes for immunosuppressants used in transplant recipients.

Class	Immunosuppressant	WHO-ATC
Anti-human T-lymphocyte Immunoglobulin, Rabbit		L04AA04
Basiliximab		L04AC02
Calcineurin inhibitors	Cyclosporine	L04AD01
	Tacrolimus	L04AD02
Inhibitors of nucleotide synthesis	Mycophenolic acid	L04AA06
	Mizoribines	L04AX
	Leflunomide	L04AA13
Antimetabolites	Azathioprine	L04AX01
mTORi	Everolimus	L04AA18
	Sirolimus	L04AA10
Corticosteroids	Methylprednisolone	H02AB04
	Prednisolone	H02AB06

mTORi, mammalian target of rapamycin inhibitor.

Supplementary Table S2. Diagnostic, procedure, and rare intractable disease codes for transplantation.

Types of organ	Code
ICD-10 code	
Kidney	Z940
Liver	Z944
Heart	Z941
Lung	Z942
Procedure code	
Kidney	R328
Liver	Q805
Liver re-transplantation	Q814, Q815
Heart	Q808
Pancreas	Q806
Lung	Q8101, Q8102
Rare intractable disease code	
Kidney	V005, V084

Liver	V013, V085
Heart	V015
Pancreas	V014, V086
Lung	V088

Supplementary Table S3. Category of immunosuppressant-related harm according to disease code (ICD-10 code) and treatment medication (WHO-ATC code).

Complications	Operational Definition	Disease code (ICD-10 code)	Treatment medication (WHO-ATC code)
**Serious infection**	Serious infection episodes were defined based on the primary diagnostic code, along with the administration of intravenous antibiotics (ATC code J) and records of hospitalization or ED visits.	**Pulmonary**<br>A37, A42.0, A48.1, B01.2, B05.2, B25.0, B39-B40, B44, B58.3, B59, B95.3, J10-J11, J12-J18, J20-J22, J85-86, U04	**IV anti-infective** (J code)
		**Musculoskeletal**<br>M00-M01, M60.0, M86	
		**Skin**<br>A36.3, A46, A60, B00-B02, B05-B06, B08-B09, B35-B36, K11.3-K12.2, L00-L05, L08, L30.3, M72.6	
		**Urinary**<br>A50-A56, N10, N30.0, N39.0, N41.0, N41.2, N41.3, N45, N70.0, N71.0, N72, N73.3, N77.1, I98.0	
		**ENT**<br>A36.0, A36.1, A36.2, A36.8- A36.9, H60.0-H60.3, H62.0-H62.4, H65.1-H65.2, H66, H68.0, H70, H94.0, J01-J06, J32, J34.0, J36-J37, J39.0-J39.1	
		**GI**<br>A00-A08, B00.8, B15, B17, B25.1, B25.8, D733, K35-K37, K57, K61, K630, K65, K670, K671, K672, K750, K770, K80-K810, K830	

		**Others:**<br>B00.5, B30, H00-H01, H03.1, H06.1, H10.5, H10.8, H13.1, H19.1-H19.2, A32.1, A39, A80-A89, B00.3-4, B01.0, B02.1, B05.1, B06.0, G00-G02, G04-G07, T82.6-T82.7, T84.5-T84.7, T85.7, A02.1, A32.7, A39.2, A39.4, A39.8, A39.9, A40-A41, B37.7, R57.2, R65.0-R65.1, A65-A69, A75-A79, I30.1, I33.0	
**Opportunistic infection**	For opportunistic infections, tuberculosis was identified using its corresponding diagnostic codes with the prescription of tuberculosis-related antibiotics (ATC code J04A). Similarly, herpes zoster was identified using corresponding diagnostic codes and prescriptions for herpes zoster-related antiviral medications (acyclovir, famciclovir, and valaciclovir). All opportunistic infections, other than tuberculosis (mycobacterial infections) and herpes virus, were categorized as ' other opportunistic infection.'	**Mycobacterial infections:**<br>A15-A19, A30-A31, K23.0, K67.3, K93.0, M01.1, M49.0, M90.0, N33.0,N74.0,N74.1	**Antimycobacterials**<br>(ATC J04A)
		**Herpes virus:**<br>A60.0, B00-02	**Antivirals**<br>(J05AB01, J05AB09, and J05AB11)
		**Viral infections:**<br>A81.2, A60.0, B17.1,B17.9,B25,B27.0,B27.1,G02.0, J17.1	
		**Bacterial infections:**<br>A02,A32,A40.3,A42,A43,A44,A481,A482,B95.3,J13	
		**Fungal infections:**<br>B37-B40, B44-B45,B59,G02.1, J17.2	
		**Parasitic infections:**<br>A072,A073,B55,B58,B78	
**Acute kidney injury**	Acute kidney injury was identified by patients who were diagnosed with acute kidney injury and had corresponding hospitalization or ED visit records.	N00.8, N00.9, N05.8, N05.9, N08, N17, N19, N28.9	-

**Hypertension emergency**	Hypertensive emergencies were characterized by patients who received intravenous antihypertensive medications (esmolol, labetalol, and nicardipine) during hospitalizations or ED visits.		**Anti-hypertensives**<br>(C07AB09, C07AG01, and C08CA04)
**Chronic kidney disease**	Chronic kidney disease was defined by the first presence of corresponding ICD-10 codes without evidence during the baseline period.	N18	
**Hypertension**	New-onset hypertension was determined by the initiation of new therapeutic agents alongside diagnostic codes in patients who did not have these conditions during the baseline period.<br>Disease worsening was identified as the initial introduction of supplementary therapeutic agents during the follow-up period in cases where patients had previously undergone treatment for the same condition before transplantation.	I10	**Alpha adrenergic blockers**<br>(C02A, C02B, C02C)<br>**Beta blockers**<br>(C07A, C07B)<br>**Calcium channel blockers**<br>(C08, C09BB, C09DB, C07FB)<br>**RAAS medications** (ATC C09)<br>**Thiazides**<br>(C03A, C09BA,C09DA,C07B,C07D,C08G)

**Diabetes mellitus**	New-onset diabetes mellitus was determined by the initiation of new therapeutic agents in patients who did not receive these agents during the baseline period.<br>Disease worsening was identified as the initial introduction of supplementary therapeutic agents during the follow-up period in cases where patients had previously undergone treatment for the same condition before transplantation.<br>(To minimize the impact of acute hyperglycemia, rapid-acting and short-acting insulins were excluded from the evaluation of diabetes mellitus.)	No disease code	**Biguanides**<br>(A10BA)<br>**Alpha glucosidase inhibitors**<br>(A10BF)<br>**Sulfonylureas**<br>(A10BB and A10BC)<br>**Thiazolidinediones**<br>(A10BG)<br>**DPP-4 inhibitors**<br>(A10BH)<br>**GLP-1 agonists**<br>(A10BJ)<br>**SGLT -2 inhibitors**<br>(A10BK)<br>**Other oral antidiabetic drugs**<br>(A10BX)<br>**Insulins**<br>(A10AB, A10AC, A10AD and A10AE)
**Dyslipidemia**	Dyslipidemia was detected by the initiation of newly prescribed lipid-lowering medications, with no anti-dyslipidemic drugs administered during the baseline period.	No disease code	**HMG CoA reductase inhibitors**<br>(C10AA)<br>**Fibrates**<br>(C10AB)<br>**Bile acid sequestrants**<br>(C10AC)

			**Other lipid-modifying agents** (C10AX)
**Osteoporosis**	Osteoporosis was defined by the first presence of corresponding ICD-10 codes without evidence during the baseline period.	M350,M80-M83,S02-S92	

ED, emergency department; GI, gastrointestinal; ENT, ear/nose/throat.

Supplementary Table S4. Frequency of immunosuppressant regimen by organ types.

Organ	Regimen	Patient-years	Patients (N)
Kidney	Tac + MPA	44,129	12,089
	CsA + MPA	6,710	1,509
	Tac	2,685	1,751
	CsA	411	289
	Tac +mTORi	40	500
Liver	Tac + MPA	18,632	5,669
	Tac	4,760	2,877
	CsA + MPA	716	284
	CsA	461	234
	Tac +mTORi	123	579
Heart	Tac + MPA	2,198	581
	CsA + MPA	250	68
	Tac + MPA + mTORi	39	108
	Tac +mTORi	26	66
	Tac	18	31
Pancreas	Tac + MPA	1,313	343
	Tac	34	40
Lung	Tac + MPA	302	193
	Tac	4	16

Tac, tacrolimus; MPA, mycophenolic acid; CsA, cyclosporine; mTORi, mammalian target of rapamycin inhibitor.

Supplementary Figure S1. A, Immunosuppressant pattern of use for maintenance regimen in liver transplant recipients. B, Kidney transplant recipients. C, Heart transplant recipients.

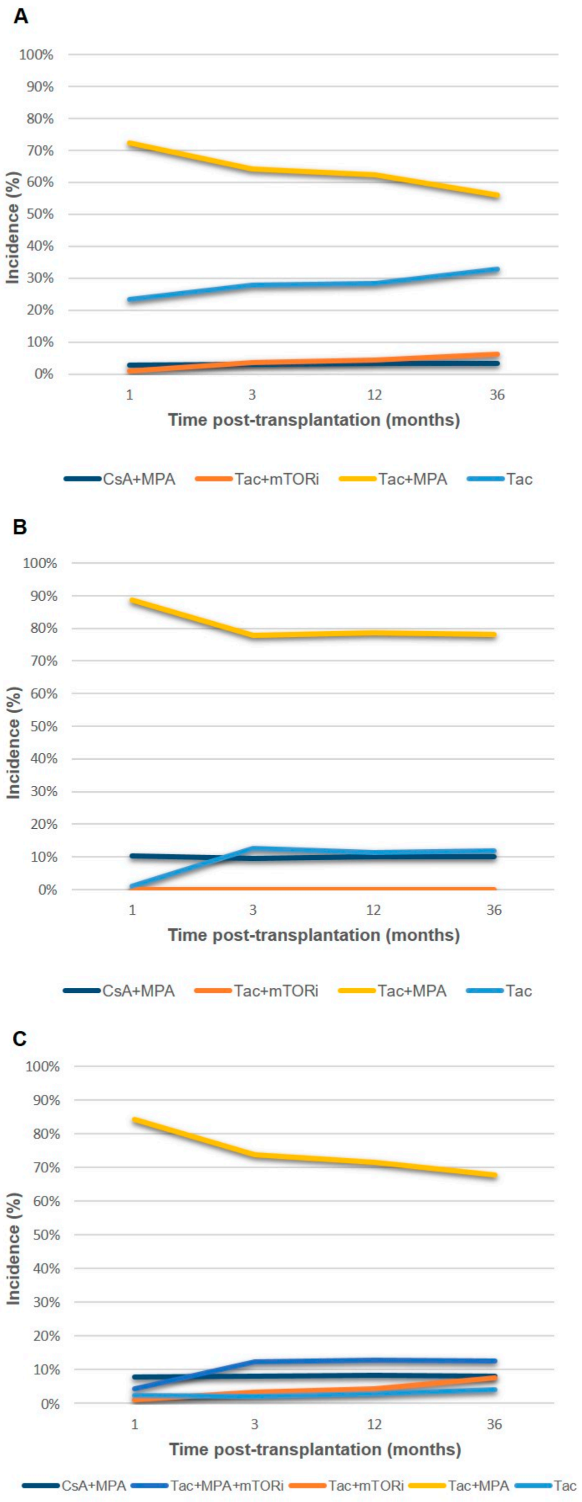

CsA, cyclosporine; MPA, mycophenolic acid; Tac, tacrolimus; mTORi, mammalian target of rapamycin inhibitor.

Supplementary Table S5. Incidence rate of complications by major regimen used over 30 days.

Organ	Regimen	Exposure (patient-years)	Total	Renal[a]	Infection[b]	Osteoporosis	Diabetes	Hypertension	Dyslipidemia
			per 1000 patient-years						
Kidney	Tac + MPA	44,129	398.9	-	121.5	51.4	55.6	112.1	58.3
	CsA + MPA	6,710	416.7	-	134.1	51.0	53.2	110.0	68.4
	Tac	2,685	496.9	-	118.8	47.7	95.7	146.8	87.9
Liver	Tac + MPA	18,632	415.1	16.6	130.4	54.3	104.7	63.9	45.2
	Tac	4,760	413.1	18.1	123.5	53.2	123.3	59.5	35.5
	CsA + MPA	716	462.2	25.1	88.0	55.9	125.7	100.5	67
Heart	Tac + MPA	2,198	462.3	8.3	187.9	50.5	85.5	80.5	49.6
	CsA + MPA	250	474.2	10.2	172.0	48.0	60.0	128.0	56.0
	Tac + MPA + mTORi	39	861.9	11	386.8	128.9	77.4	128.9	128.9
Pancreas	Tac + MPA	1,313	332.3	15.6	139.3	60.9	34.3	60.9	21.3
	Tac	34	305.7	71.2	58.6	0	0	146.6	29.3
Lung	Tac + MPA	302	599.6	41.0	238.0	112.4	79.3	62.8	66.1

[a]Renal dysfunction was defined by composite of chronic kidney disease and acute kidney injury.

[b]Infection was defined by composite of serious infection and opportunistic infection.

Tac, tacrolimus; MPA, mycophenolic acid; CsA, cyclosporine; mTORi, mammalian target of rapamycin inhibito
